# Barriers to utilization of primary healthcare services: a systematic review of experiences in ten selected countries

**DOI:** 10.3389/ijph.2026.1609249

**Published:** 2026-05-12

**Authors:** Hakimeh Mostafavi, Efat Mohamadi, Amin Mohammadi, Ahad Bakhtiari, Jawad Jafarzadeh, Alireza Olyaeemanesh, Amirhossein Takian

**Affiliations:** 1 Health Equity Research Centre (HERC), Tehran University of Medical Sciences (TUMS)., Tehran, Iran; 2 Iranian Research Network for Social Determinants of Health (IRNSDH), Tehran, Iran; 3 Department of Health Policy, Management and Economics, School of Public Health, Tehran University of Medical Sciences (TUMS), Tehran, Iran; 4 Centre of Excellence for Global Health, Department of Global Health & Public Policy, Tehran University of Medical Sciences (TUMS), Tehran, Iran

**Keywords:** access, health for all, Levesque’s framework, primary health care (PHC), utilization

## Abstract

**Objectives:**

This study aimed to identify the reasons for inadequate use of PHC services to mitigate the barriers to utilization of PHC services in Saudi Arabia, Oman, Türkiye, Pakistan, Iraq, Thailand, China, India, Egypt, and Iran.

**Methods:**

This is a systematic review that synthesized the findings of original studies focused on the barriers to the utilization of PHC services. We searched the MEDLINE, Scopus, and Google Scholar databases from February 1, 2000, to December 29, 2023, in English. We conducted content analysis facilitated by MAXQDA-10 software drawn upon Levesque’s framework.

**Results:**

The screening of articles was conducted in accordance with the Preferred Reporting Items for Systematic Reviews and Meta-Analyses (PRISMA) checklist. The initial search retrieved 1,613 results, of which we included 29 studies. In terms of methodology, 17 studies used quantitative methods, eight had qualitative approaches, and the remaining studies utilized mixed or other methods. Among the five groups of identified barriers, ability to perceive, ability to reach, and ability to pay were found to be noteworthy barriers that should be considered by health policymakers.

**Conclusion:**

Although the main barriers to inadequate use of PHC are related to people, raising awareness about the need for PHC, improving literacy through understandable training, and establishing mobile facilities in remote areas are appropriate strategies for increasing the use of PHC services.

## Introduction

Access to healthcare services is a fundamental pillar of national progress, as it promotes, maintains, and ensures the wellbeing of the population. As the first level of basic care in many countries, PHC is designed to deliver essential and low-cost healthcare services to the public at the community level [1]. The Alma-Ata Declaration by the World Health Organization (WHO) in 1978 manifested a milestone in public health, identifying PHC as the foundation for achieving the goal of “Health for All” [2]. In response, many countries began to prioritize the provision of essential public health services to their populations through the establishment of PHC networks.

In 2018, celebrating the 40th anniversary of the Alma-Ata Declaration on PHC in 1978, the Astana Declaration emphasized that PHC serves as a person’s first point of contact when people seek healthcare, dealing with most problems, and acting as the lever of the health system [3]. The 45th Anniversary of the historical PHC declaration in Astana-Kazakhstan underscored the identification of policies and practices to future-proof PHC transformation for moving towards Universal Health Coverage (UHC), for greater resilience in the face of emergencies, and for better health and wellbeing [4].

Despite efforts for the ideal use of PHC, countries have been facing various obstacles to appropriate use of PHC facilities, associated with their socio-economic status, as well as cultural factors and population values [5]. For instance, in Türkiye, the PHC system operates under a family medicine model, providing free preventive care, vaccination, and maternal and child health services. However, disparities exist between urban and rural areas, and family physicians often feel overburdened due to the high number of services they have to perform [6]. Similarly, Saudi Arabia has improved its PHC system with free services, but it still struggles with patients bypassing PHC centers for hospitals and with high turnover among healthcare workers [7]. In India, the PHC system is strengthened by the National Health Mission. Although it provides essential services, it suffers from inadequate infrastructure, long waiting times, and uneven quality of care [8]. In China, reforms have bolstered PHC services, yet challenges persist, including poor coordination and low trust in the system, especially between urban and rural areas [9]. Thailand and Pakistan have also reported mixed experiences with their PHC systems. Despite its undeniable role in achieving UHC, resource imbalances, healthcare workers’ burnout, and lack of mental health services remain major concerns [10]. In Egypt, overcrowded and under-resourced facilities hinder the PHC system’s ability to meet the population’s needs [11]. War-torn Iraq faces severe damage to its PHC infrastructure and health worker shortages [12]. Oman has a relatively strong PHC system but struggles with healthcare worker shortages and the rising burden of NCDs [13]. Iran’s PHC network is well-established, though challenges include workforce shortages, inefficient coordination, and an aging population that strains resources [1, 14].

Despite global and national efforts to strengthen PHC systems, a comprehensive understanding of the barriers to the utilization of PHC services remains fragmented. Studies have highlighted challenges in specific regions or aspects of PHC, such as infrastructure deficits, workforce shortages, or disparities between urban and rural areas [15]. However, a systematic synthesis of these barriers across different socio-economic, cultural, and political contexts is lacking. This gap limits the ability of policymakers and health managers to develop adaptable, evidence-based strategies for improving PHC utilization globally.

This study fills this gap by systematically reviewing the experiences of ten countries, including Saudi Arabia, Oman, Türkiye, Pakistan, Iraq, Thailand, China, India, Egypt, and Iran, providing a comparative analysis of the obstacles these nations face in the utilization of PHC services.

## Methods

We conducted this systematic review following the guidelines of the Preferred Reporting Items for Systematic Reviews and Meta-Analyses (PRISMA) [16]. The study was approved by the ethical committee of Research Ethics Committees of Tehran University of Medical Sciences (TUMS), under the ethical code of IR.TUMS.SPH.REC.1402.143. The protocol stated the research questions, objectives, exclusion and inclusion criteria, and other preparatory details for this systematic review. Initially, we developed a preliminary list of search terms and searched MEDLINE, Scopus, and Google Scholar to assess the relevance of the results. To further refine the search terms, we manually examined reference lists from several relevant studies and similar reviews. Subsequently, we adjusted search query to align with the subject headings specific to each database. The complete search strategy is detailed in Supplementary File 1.

**FIGURE 1:**
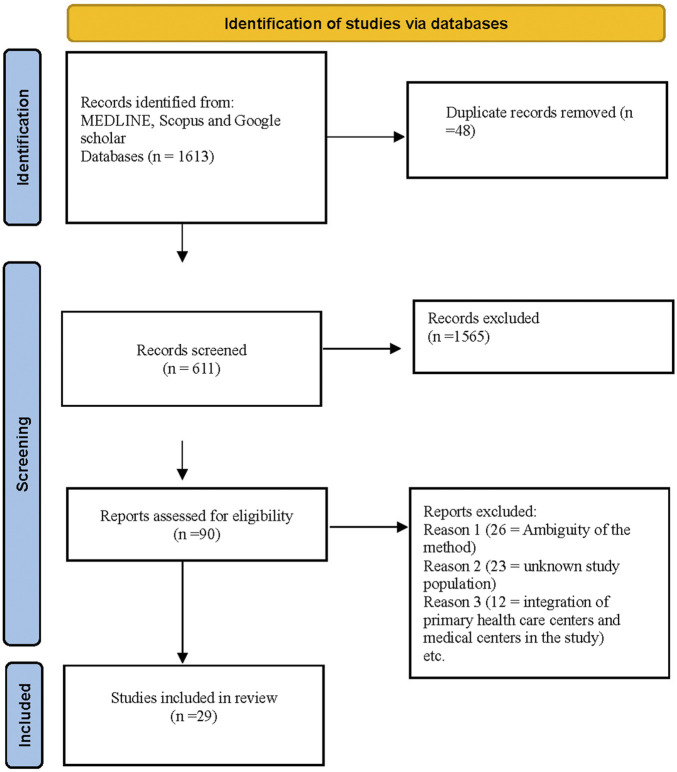
Prisma flow chart of included studies (Multiple Countries, 2001-2023).

Two authors (AM and AB) conducted the database searches, while two other authors (HM and EM) independently selected relevant studies and extracted the data. In cases of disagreement regarding study selection, a third team member (AO) was consulted to reach a consensus. For data extraction, we created a preliminary data extraction form to systematically record information from the finalized studies. Data were extracted at two levels: primary information, which included the authors’ names, study design, language of the study, and year of publication, and secondary information, which encompassed details pertinent to the study’s objectives. The process of study selection and data extraction is illustrated in the PRISMA flowchart, presented in Figure 1.

### Study eligibility criteria

We utilized a PEO (Population, Exposure, and Outcome) framework and constructed a detailed guide to the studies to be included. Our population included Iran, Thailand, Iraq, Saudi Arabia, Egypt, China, Pakistan, Türkiye, Oman, and India. Exposure included utilization of primary healthcare services, and our outcomes assessed the barriers utilization of PHC services based on the Levesque’s framework.

### Inclusion criteria

Studies included in this review were required to be published in English and conducted within PHC settings of 10 selected countries: Türkiye, Saudi Arabia, India, China, Thailand, Pakistan, Egypt, Iraq, Oman, and Iran. In selecting the 10 countries, we considered both methodological and contextual factors. First, the chosen countries share significant socioeconomic and health system challenges that directly impact the accessibility and effectiveness of PHC services. These include limited healthcare infrastructure, rural-urban health disparities, and financial constraints within their health sectors. Second, most of the chosen countries are characterized by a similar level of economic development, where low to middle-income statuses create systemic barriers such as underfunding, inadequate staffing, and insufficient resources in primary care. Third, the selected countries exhibit demographic similarities, e.g., growing aging populations, leading to increased demand for PHC services. They are also experiencing a double burden of disease, with a high prevalence of both communicable and non-communicable diseases, further straining their PHC systems. Methodologically, this selection allows for a comparative analysis across different yet comparable health systems, which may provide broader insights into shared barriers and inform more globally relevant solutions. By choosing countries with these overlapping characteristics, the study aims to produce findings that are generalizable to other similar contexts.

### Exclusion criteria

Studies that did not indicate the barriers of utilization of PHC, conference papers, meeting abstracts, review papers, books, and non-English papers were excluded. We also excluded studies that only examined barriers to healthcare in general (not primary care).

### Validity of the review process

To ensure the validity of the selection process, two members of the research team (AB and HM) conducted the review. After an initial screening process, which involved the removal of duplicates and unrelated studies, the two researchers (AT and AO) independently assessed the remaining articles for relevance to the topic and availability of extractable information. Any disagreements were resolved through consensus between the two reviewers.

### Quality assessment

We used two different tools to evaluate the quality of studies. The Critical Appraisal Skills Program (CASP) checklist is widely recognized for assessing qualitative research, focusing on aspects such as study rigor, credibility, and relevance of findings [17] In contrast, the Effective Public Health Practice Project (EPHPP) tool is tailored for evaluating quantitative studies, including randomized and non-randomized designs, by systematically examining selection bias, study design, confounders, blinding, data collection methods, and withdrawals/dropouts [18]. Using these two complementary tools allowed us to rigorously and appropriately assess the methodological quality of both qualitative and quantitative studies in our review.

### Analysis

We conducted analysis by initially grouping together frequently reported outcomes through content analysis, and then we performed the final analysis using Levesque’s framework, which helped us categorize the findings clearly and avoid inconsistency and bias in our research. This framework outlines five key abilities of populations that influence their access to and use of PHC services [19], i.e., ability to perceive, ability to seek, ability to reach, ability to pay, and ability to engage.

## Results

Our search retrieved 1,613 results, of which 29 studies met the inclusion criteria. The literature search process is represented in the flowchart adapted from the PRISMA guidelines (Figure 1). Our analysis revealed various barriers contributing to the inadequate utilization of first-level health services. The quality assessment of studies, characteristics, and outcomes of the 29 included studies are summarized in Table 1. Based on the quality assessment of studies, 25 articles were classified as high quality, while four were deemed of moderate quality. India had the highest representation in the review, with nine studies, whereas Egypt had the lowest, with only one study. In terms of methodology, 17 studies used quantitative methods, eight adopted qualitative approaches, and the remaining studies utilized mixed or other methodologies.

**TABLE 1:** Descriptive characteristics of studies and their quality assessment (Multiple Countries, 2001-2023).

Study (author)	Study title	Country	Year	Study design	Population	Quality assessment	Ref
Duygu Ayhan Baser	Views and experiences of family physicians about Syrian refugee patients in Türkiye: Qualitative research	Tukey	2011	Qualitative study	Family physicians	High	[20]
Ghada Wahby Elhady	Postnatal care in rural Egypt: Perspectives of women and healthcare providers	Egypt	2014	An exploratory cross-sectional study	Women and healthcare providers	High	[21]
Ghadah Alfaqeeh	Access and utilisation of primary healthcare services comparing urban and rural areas of Riyadh Providence, Kingdom of Saudi Arabia	Saudi Arabia	2017	National survey	Rural and urban population	High	[22]
Gilbert Burnham	Perceptions and utilization of primary healthcare services in Iraq: Findings from a national household survey	Iraq	2011	Qualitative study	Rural and urban population	High	[23]
Lemmese AlWatban	Are physicians creating a barrier to pre-conception care access? A qualitative study exploring patients’ experiences and perspectives around pre-conception care	Saudi Arabia	2019–2020	Qualitative study	Patients	Moderate	[24]
Nayeb Fadaei Dehcheshmeh	Challenges of middle-aged men in utilizing new health services from primary healthcare providers’ perspective: a Qualitative study	Iran	2019	Qualitative study	Primary healthcare providers	High	[25]
Qais Alemie	Determinants of healthcare services utilization among first generation Afghan migrants in Istanbul	Türkiye	2017	Cross-sectional study	Afghan migrant	High	[26]
Ahmed Nasser Al-luhaym	Factors associated with access to the Saudi primary healthcare in Light of vision 2030	Saudi Arabia	2023	Cross-sectional study	Patients	High	[27]
Banerjee SK	An exploration of the socio-economic profile of women and costs of receiving abortion services at public health facilities of Madhya Pradesh, India	India	2014	Qualitative study	Women	High	[28]
Tazeen Hasan Jafar	Access to CKD care in rural communities of India: A qualitative study exploring the barriers and potential facilitators	India	2020	Cross-sectional study	Rural communities	Moderate	[29]
Tej Ram Jat	Factors affecting the use of maternal health services in Madhya Pradesh state of India: A multilevel analysis	India	2011	National survey- cross-sectional study	Women	High	[30]
Yinzi Jin	Impact of health workforce availability on healthcare seeking behavior of patients with diabetes mellitus in China	China	2017	National survey- cross-sectional study	Patients with diabetes mellitus	High	[31]
Yuehua Chen	Utilization and out-of-pocket expenses of primary care among the multimorbid elderly in China: A two-part model with nationally representative data	China	2022	Cross-sectional study	Multimorbid elderly	High	[32]
Jean-Frédéric Levesque	Patient-centered access to healthcare: conceptualizing access at the interface of health systems and populations	India	2006	Cross-sectional study	Urban population	High	[19]
Changle Li	Bypassing primary care facilities: Health-seeking behavior of middle age and older adults in China	China	2021	Cross-sectional study- logistic models	Rural and urban population	Moderate	[33]
Li Li	Socioeconomic determinants are associated with the utilization and outcomes of active surveillance or watchful waiting in favorable-risk prostate cancer	China	2020	National survey- cross-sectional study	Aged individuals	High	[34]
S Siddiqi	Does contracting out lead to improvement in service volumes at primary and secondary health services? Evidence from rural districts of Sindh, Pakistan	Pakistan	2001	Qualitative study	Rural and urban population	High	[35]
Vijay Silan	Determinants of underutilisation of free delivery services in an area with high institutional delivery rate: A qualitative study	India	2014	Qualitative study	Social activists	High	[36]
Kumar S	Implementation of a large-scale breast cancer early detection program in a resource-constrained setting: real-world experiences from 2 large states in India	India	2023	Cross-sectional study	Rural population	High	[37]
Abha Tewari	Process evaluation of the systematic medical appraisal, referral and treatment (SMART) mental health project in rural India	India	2017	Mixed method	Rural population	High	[38]
Li X	Quality of primary healthcare in China: challenges and recommendations	China	2021	Cross-sectional study	Hypertensive patients	Moderate	[39]
Beibei Yuan	Disadvantaged populations in maternal health in China who and why?	China	2014	Review	Women	High	[40]
Karoline Kragelund Nielsen	Factors influencing timely initiation and completion of gestational diabetes mellitus screening and diagnosis - a qualitative study from Tamil Nadu, India	India	2017	Qualitative study	Pregnant women- health providers	High	[41]
Hadi Karimi Nodehi	Strategic analysis of community participation in primary healthcare in iran and presentation of promotion strategies using internal and external environment assessment techniques	Iran	2021	Mixed method	PHC centers	High	[42]
S Reshadat	Spatial accessibility of the population to urban health centres in Kermanshah, Islamic Republic of Iran: A geographic information systems analysis	Iran	2015	Quantitative study-	Urban population	Moderate	[43]
Atif Riaz	Perceived barriers to utilizing maternal and neonatal health services in contracted-out versus government-managed health facilities in the rural districts of Pakistan	Pakistan	2015	Community-based qualitative exploratory study	Rural women	Moderate	[44]
Rattanakarun Rojjananukulpong	Disparities in physical accessibility among rural thais under universal health coverage	Thailand	2021	Cross-sectional study	Rural population	High	[45]
Jayanti Saha	The Pattern of Morbidity and access to healthcare service in the Riverine Flood-prone villages of Assam, India	India	2023	Mixed method	Rural population	High	[46]
Hui Sang	Is low cost really conducive to primary care utilisation: An empirical analysis of community health centers in China	China	2021	Cross-sectional study	Urban population	High	[47]

### Results according to Levesque’s conceptual framework

According to Levesque’s conceptual framework, we classified the barriers into five themes, i.e., ability to perceive barriers, ability to seek barriers, ability to reach barriers, ability to pay barriers, and ability to engage barriers. Our analysis identified ability to perceive, ability to reach, and ability to pay as very significant barriers. Table 2 shows the frequency of barriers, followed by an explanation for each of the themes.

**TABLE 2:** Themes, sub-themes, and their frequency in 29 studies (Multiple Countries, 2001-2023).

Themes	Sub-themes	Number
Ability to seek	Male pride individual values ​​based on men not needing health services, Ethnic and tribal values ​​in rural areas lack of belief in receiving services from female employees Lack of belief in receiving services from men	11
Ability to perceive	Lack of belief in the importance of care health literacy Unfamiliarity with effective role of oneself religious beliefs	23
Ability to reach	Distance from the center, lack of public transportation, cost of transportation Uneven path Location of the center in a remote area	20
Ability to engage	Language comprehension problem, lack of family support, Lack of social support	7
Ability to pay	Low income, poor living conditions, High drug costs High visit costs Financial problems	18

### Barriers to ability to seek

We identified various factors influencing individuals’ ability to seek services at PHC centers. For instance, religious beliefs and adherence to religious practices can prevent women from visiting PHC centers that lack female staff [19, 48]. Cultural values [49], specific cultural attitudes, beliefs, and misunderstandings, and shyness of women are other reasons for little utilization of PHC centers [50]. Being among ethnic minorities [7] and not believing in receiving services from female staff may result in not using the health services by men [51]. Other studies [6, 52] mentioned old age as a factor that causes the failure to receive required health services.

### Barriers to the ability to reach

Frequent replacements of PHC centers may prevent refugees from accessing essential PHC services [48]. Further, long distances to health centers, requiring rural populations to walk for over an hour, discouraged them from utilizing these services. The need to cover transportation costs, along with the lack of suitable transportation options, are additional factors contributing to the underutilization of health services [49].

### Barriers to ability to perceive

Studies have identified various perception-related factors that hinder the utilization of health services. For example, a lack of understanding about the importance and necessity of health services prevented individuals from visiting PHC centers [53]. Additionally, low health literacy has been linked to insufficient use of health services. Furthermore, misconceptions and beliefs regarding the value of health services, particularly among men, may contribute to underutilization by them and their families [51]. Lastly, the perceived inadequacy of personnel’s qualifications is considered as a significant factor in the insufficient use of PHC centers.

### Barriers to ability to pay

Various factors affect the ability to pay and consequently utilization of PHC services. Not affording to pay for necessary medicines [54] or doctor’s fees, as well as the families’ low income and other financial limitations, may also affect the use of PHC services.

### Barriers to ability to engage

Different factors result in people’s low engagement in utilizing the PHC services. For example, a study indicated that Syrian refugees were unable to effectively follow medical instructions because they do not understand the Turkish language, and translators were unavailable at health service centers. As a result, even when services are accessible, language difficulties may prevent effective use of health services. Another study reported that lack of support from family members and social organizations hampers patients’ ability to complete their treatment in the Tamil Nadu region of India.

### Health system barriers

A review of studies showed that insufficient female physicians in PHC centers prevent women from visiting these centers to seek necessary health services. Studies pointed to insufficient medical facilities and equipment, limited laboratory facilities, and old medical facilities as the main reasons for insufficient referral of people to PHC centers [55], ineffective communication of physicians with mothers [56], and insufficient human resources in PHC centers as the reasons behind underutilization of PHC services [21].

## Discussion

In this systematic review, we aimed to identify multifaceted barriers influencing the utilization of PHC services service in including Saudi Arabia, Oman, Türkiye, Pakistan, Iraq, Thailand, China, India, Egypt, and Iran through Levesque’s framework to understanding the individual-level barriers and highlighting broader systemic challenges that need to be addressed to improve PHC utilization. The selection of the ten countries was informed by regional, socioeconomic, and health system considerations. The countries represent the Middle East, North Africa, and Asia—regions where primary healthcare (PHC) has been prioritized but continues to face persistent challenges. They also span a broad range of contexts, from high-income (e.g., Saudi Arabia, Oman) to middle-income (e.g., Iran, Türkiye, Egypt, Thailand) and lower-income or conflict-affected settings (e.g., Pakistan, Iraq), enabling comparative analysis across different economic and political conditions.

All selected countries face a dual burden of disease, with ongoing communicable diseases alongside rapidly rising noncommunicable diseases (NCDs). In several cases, including Iran, Egypt, and Saudi Arabia, NCDs account for over 70% of total mortality, increasing pressure on PHC systems. Documented challenges—such as infrastructure limitations, workforce shortages, bypassing of PHC, and urban–rural disparities—further justify the comparative approach. Accordingly, this review provides cross-contextual insights into PHC system resilience and equity, while its findings should be interpreted as broad rather than country-specific.

The review highlighted important perception-related barriers to PHC utilization, including low health literacy, misconceptions about the necessity of health services, and a lack of awareness regarding personal health responsibilities and misunderstandings of the physicians’ recommendations because of language problems. Particularly in rural and underserved areas, enhancing health literacy through community-based programs could foster better health-seeking behavior and mitigate the impact of these barriers. Similarly, cultural beliefs have been reported to influence healthcare-seeking behavior at the PHC level in Pakistan [57]. Similar to our study, Adetola Emmanuel Babalola (2025), in their study, showed that the use of local languages in healthcare delivery improves compliance with medical instructions and health improvement [58]. Contrary to our findings, another study in Iran showed that health literacy and healthcare utilization were not statistically associated [59]. Belief and religious factors appeared as prominent barriers, particularly for women in conservative societies. In other words, although countries always try to provide accessible health services for their population, traditional values ​​and beliefs are still a big obstacle in some settings. In such a situation, it might be useful to employ local people to align the provision of health services not in conflict with people’s beliefs and values. These findings are similar to findings of the other studies in Nepal and Bangladesh [60, 61].

Barriers to reaching PHC centers were the second significant barrier. Geographic barriers, i.e., long distances to health facilities and inadequate transportation, were recurrent themes in the studies reviewed. This is particularly problematic in rural and remote areas, where populations are often required to travel long distances, sometimes on foot, to access health services. A study in Mozambique showed that difficult access to PHC centers has a high impact on their utilization, meaning that people are more likely to visit health centers if public transportation is available [55]. Addressing these barriers requires investments in healthcare infrastructure and transportation networks, particularly in regions with dispersed populations. Placement of PHC centers and mobile health clinics could alleviate some of these access issues. Similar to our findings, Nazia Shahzadi indicated that leveraging technology and innovative healthcare delivery models can help overcome the unique challenges faced by rural populations [62]. However, transportation infrastructure expansion had both negative and positive effects on spatial access to healthcare for different villages. Using a complete georeferenced census of community health posts in Niger and other high-resolution spatial datasets increased the use of services in these centers [63].

Lastly, financial constraints are another significant barrier to PHC utilization, which makes it difficult for many individuals to afford necessary medications or PHC services. Similar to our findings, an Australian study showed that affordability was perceived as a substantial barrier to the use of PHC services [64]. Although PHC in most countries is free for all, economic problems such as restricted employment opportunities, multiple health problems of family members, and high costs of transportation may cause inadequate use of PHC services. The findings suggest that economic barriers are pervasive across the countries studied, affecting both urban and rural populations, with a more significant impact on rural areas.

Beyond individual-level barriers, our review identified several systemic issues that may limit the effectiveness of PHC services, i.e., an insufficient number of female healthcare providers, inadequate medical facilities, and poor communication between healthcare workers and patients. Others indicated that unavailability of the usual doctor, waiting times and doctors not accepting new patients were the most important causes of unmet needs for care in Quebec province- Canada [65]. Addressing these issues requires systemic reforms, including enhancing healthcare worker training, improving facility infrastructure, and ensuring a sufficient and gender-diverse healthcare workforce.

This review has several strengths, including the use of a systematic search strategy, adherence to PRISMA guidelines, and a comprehensive synthesis of evidence on primary healthcare service delivery in crisis and conflict settings. However, several limitations should be acknowledged. First, the review included only English-language, peer-reviewed publications, which may have led to the exclusion of relevant studies published in other languages or in the grey literature. Second, the included studies covered highly heterogeneous country contexts in terms of economic conditions, social structures, and health system organization, which may limit comparability across studies. Third, variations in study design, outcome definitions, and reporting quality constrained the ability to conduct more granular or country-specific analyses. These limitations should be considered when interpreting the findings and assessing their applicability to specific national or subnational contexts.

Our findings suggest several policy and practice implications. First, there is a need for comprehensive health education programs that can improve health literacy and address misconceptions about health services. Second, policies need to prioritize the recruitment and retention of female healthcare workers, particularly in conservative regions, to improve access for women. Third, expanding health infrastructure and ensuring the availability of affordable transportation options are crucial for enhancing physical access to health services. Lastly, addressing financial barriers through subsidized healthcare costs and improved insurance coverage could mitigate the economic burden on individuals seeking care.

Based on the findings, we suggest some recommendations and future directions, including conducting longitudinal and multi-country studies to better understand how socioeconomic, cultural, and political contexts shape PHC utilization over time. Exploring the impact of digital health solutions, such as telemedicine and mobile health applications, in bridging urban–rural disparities. Assessing the cost-effectiveness of interventions designed to reduce barriers, particularly in resource-limited settings. Investigating gender and equity dimensions in PHC access, ensuring that vulnerable populations—including women, migrants, and those living in conflict-affected areas—are adequately represented in future studies.

PHC is the only feasible media to reach UHC in many countries. Yet, several barriers have hampered appropriate utilization of PHC services in many settings, particularly in LMICs. We draw policymakers’ and healthcare providers’ attention to these barriers, particularly those related to the ability to perceive, the ability to reach and the ability to pay for designing and implementing the interventions. Designing educational programs to increase people’s understanding of the importance of PHC, expanding public transportation or providing mobile health services, and negotiating with insurance organizations to cover high-demand health services may result in willingness to use PHC.
